# Effect of negative air ions on the potential for bacterial contamination of plastic medical equipment

**DOI:** 10.1186/1471-2334-10-92

**Published:** 2010-04-12

**Authors:** Simon J Shepherd, Clive B Beggs, Caroline F Smith, Kevin G Kerr, Catherine J Noakes, P Andrew Sleigh

**Affiliations:** 1 Bradford Infection Group, School of Engineering, Design and Technology, University of Bradford, Bradford, BD7 1DP, UK; 2 Harrogate and District NHS Foundation Trust, Harrogate District Hospital, Lancaster Park Road, Harrogate, HG2 7SX, UK; 3 Pathogen Control Engineering Institute, School of Civil Engineering, University of Leeds, Leeds, LS2 9JT, UK

## Abstract

**Background:**

In recent years there has been renewed interest in the use of air ionizers to control the spread of infection in hospitals and a number of researchers have investigated the biocidal action of ions in both air and nitrogen. By comparison, the physical action of air ions on bacterial dissemination and deposition has largely been ignored. However, there is clinical evidence that air ions might play an important role in preventing the transmission of *Acinetobacter *infection. Although the reasons for this are unclear, it is hypothesized that a physical effect may be responsible: the production of air ions may negatively charge items of plastic medical equipment so that they repel, rather than attract, airborne bacteria. By negatively charging both particles in the air and items of plastic equipment, the ionizers minimize electrostatic deposition on these items. In so doing they may help to interrupt the transmission *of Acinetobacter *infection in certain healthcare settings such as intensive care units.

**Methods:**

A study was undertaken in a mechanically ventilated room under ambient conditions to accurately measure changes in surface potential exhibited by items of plastic medical equipment in the presence of negative air ions. Plastic items were suspended on nylon threads, either in free space or in contact with a table surface, and exposed to negative ions produced by an air ionizer. The charge build-up on the specimens was measured using an electric field mill while the ion concentration in the room air was recorded using a portable ion counter.

**Results:**

The results of the study demonstrated that common items of equipment such as ventilator tubes rapidly developed a large negative charge (i.e. generally >-100V) in the presence of a negative air ionizer. While most items of equipment tested behaved in a similar manner to this, one item, a box from a urological collection and monitoring system (the only item made from styrene acrylonitrile), did however develop a positive charge in the presence of the ionizer.

**Conclusion:**

The findings of the study suggest that the action of negative air ionizers significantly alters the electrostatic landscape of the clinical environment, and that this has the potential to cause any *Acinetobacter*-bearing particles in the air to be strongly repelled from some plastic surfaces and attracted to others. In so doing, this may prevent critical items of equipment from becoming contaminated with the bacterium.

## Background

In recent years there has been renewed interest in the use of air ionizers to control the spread of infection in hospitals [1] and a number of researchers have investigated the biocidal action of ions in both air [2-9] and nitrogen [2,10]. While the physical action of air ions on particles has received some attention [11-14] the role of ionizers in bacterial dissemination and deposition has largely been ignored. However, there is evidence from a clinical setting that air ions might play an important role in preventing the transmission of some infections. In a trial conducted on an intensive care unit (ICU), Kerr *et al*. [1] found that the presence of negative air ionizers was associated with a significant decrease *Acinetobacter *infection or patient colonization, despite the fact that numbers of environmental isolates of *Acinetobacter *spp increased. This suggests that the observed reduction in *Acinetobacter *infection or patient colonization was probably due to physical effects rather than any bactericidal phenomena. Although the reasons for the results observed by Kerr *et al*. [1] are unclear, it is hypothesized that the air ions may have negatively charged items of plastic medical equipment in the ward, such as patient ventilator tubes, so that they repelled, rather than attracted, airborne bacteria. Widespread aerial dissemination of bacteria is thought to occur within the clinical environment [15,16] due to activities such as bed making, and this has been implicated in a number of outbreaks of *Acinetobacter *infection [17-19]. In the course of normal operation, many items of plastic equipment naturally acquire an electric charge [20], and this can promote electrostatic precipitation of bacteria, carrying an opposed charge, from the air. By negatively charging both particles in the air and items of plastic equipment, the ionizers potentially minimize electrostatic deposition on these surfaces. In order to test this hypothesis we designed the experimental study to investigate the behaviour of items of plastic medical equipment in the presence of negative air ions and to assess the likely impact on the precipitation of airborne particles.

## Methods

**Figure 1:**
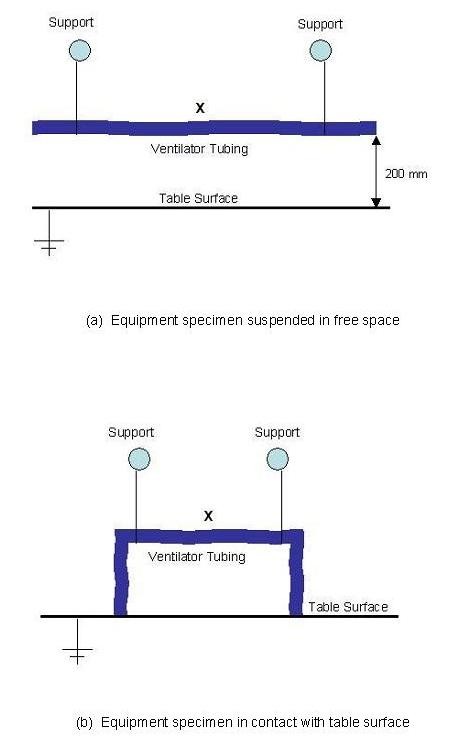
**General arrangement of specimens during experimentation (X marks the location of static charge measurements)**. (a) Equipment specimen suspended in free space. (b) Equipment specimen in contact with table surface.

The study was undertaken in a mechanically ventilated room (dimensions 3 × 2 × 2.5 m high) under ambient conditions. The aim of the study was to measure accurately changes in surface potential exhibited by items of plastic medical equipment in the presence of negative air ions. During experimentation plastic items were suspended on nylon threads, either in free space or in contact with a table surface (see Figure 1), and exposed to negative ions produced by a direct current unipoler air ionizer (WM 120, Air Ion Technologies Limited, New Milton, UK) with an electrode potential of -5 kV. Items examined were: ventilator tubing (Breathing System 2000, Intersurgical Ltd, Wokingham, UK); a ventilator mask (SealFlex™ single port, Caradyne Ltd, Dublin, Ireland); nebulizer tubing (MicroMist™, Hudson Respiratory Care Inc, North Carolina, USA); Unometer™ measuring chamber and collection tubing (both Unomedical Ltd, Redditch, UK) and a disposable apron (BPI Healthcare, Heanor, UK). The charge build-up on the specimens was measured using an electric field mill (JCI 140, John Chubb Instrumentation, Cheltenham, UK), which was located perpendicular to the specimen surface at a distance of 100 mm. During the various experiments the ion concentration in the room air was recorded using a portable ion counter (Air Ion Counter IC 1000, Ion Trading, Tokyo, Japan). The air temperature and humidity in the room space were also recorded.

Table 1 lists the items tested, together with their dimensional and material characteristics. Each item of equipment was tested for a total of 3600 s - an initial period of 600 s in which the ionizer was not in operation, a second period of 2200 s in which the ionizer was in operation, and a final period of 800 s in which the ionizer was again inoperative. The surface potential was recorded every 30 s. This regime was selected because it allowed the transient charge build-up and decay to be characterized for each specimen item of equipment.

**Table 1:** Characteristics of the items of equipment used in the study

Item of Equipment	Description	Component Tested	Component Material	Characteristic Dimension	Relative Electrical Permittivity @ 1 Mhz
Ventilator tubing	Parallel twin plastic ventilator tubes (1.6 m long)	Centre portion of tube	Low density polyethylene (LDPE)	25 mm external diameter tube	2.2 - 2.35

Ventilator Mask	Single port mask with attachment for ventilator tubing	Mask	Cushion Thermoplastic elastomer (TPE)	Height: 110 mm Width: 85 mm Depth: 42 mm	Unknown

Nebulizer tubing	Nebulizer with reservoir tubing	Reservoir tubing	Polyvinylchloride (PVC)	6 mm external diameter tube	2.8

Unometer™ box and tubing	Urological collection and monitoring system with collection bag	Plastic measuring box	Styrene acrylonitrile (SAN)	Height: 105 mm Width: 180 mm Depth: 45 mm	2.55 - 2.95

Unometer™ box and tubing	Urological collection and monitoring system with collection bag	Tubing	Polyvinylchloride (PVC)	9 mm external diameter tube	2.8

Disposable Apron	Plastic disposable Apron	Apron	Polyethylene (PE)	265 mm × 275 mm	2.3

**Figure 2:**
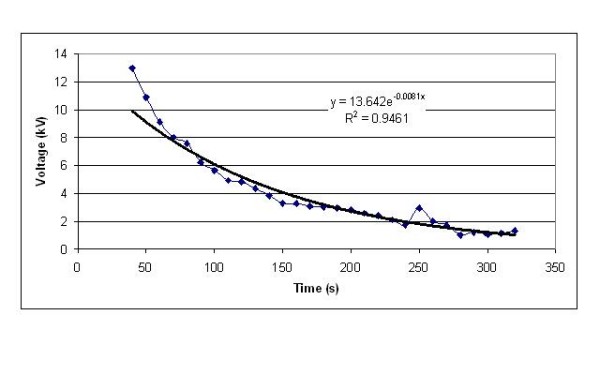
**Charge decay in a ventilator tube suspended with its ends in contact with an earthed table**.

In order to characterize the electrical properties of the test apparatus described above, we arranged a LDPE ventilator tube as shown in Figure 1(b), so that its ends were in contact with the table top. We then charged the tube by rubbing it with a cloth and recorded the subsequent discharge over a 5 minute period. The results of this experiment are presented in Figure 2. From this it can be seen that the charge decay is exponential, with a *CR *constant of approximately 140 seconds, which corresponds to a leakage resistance of about 10^13 ^Ω and a capacitance of 14 pF. When the procedure was repeated for the ventilator tubing suspended in free space (i.e. without contact with the table), no significant discharge was recorded. In order to calculate the strength of the electric field around the ventilator tubing equation 1 was used.

Where, *q*, is the charge on the surface of the plastic tube, *ε*_0 _is the *permittivity of free space *which is approximately 8.854 × 10^-12 ^F/m, *ε*_r _is the *relative permittivity *of the tubing material and *r *is the radius of the tube.

## Results

The ion count and air condition data recorded during the various experiments are presented in Table 2. From these data it can be seen that under normal conditions (i.e. without the ionizer in operation), the negative air ion count in the test room was generally <1000 ions/cm^3^. However, when the negative ion generator was in operation, the negative ion count rapidly increased to stabilize at values in the range 28800 - 85600 ions/cm^3^. During the experiments conditions in the test room were recorded and found to be stable with relatively little variation in air temperature or humidity.

**Table 2:** Mean air ion counts and air conditions recorded during the various experiments

Experiment	Negative ion count (ions/cm^3^)	Negative ion count (ions/cm^3^)	Negative ion count (ions/cm^3^)	Average air condition
	**Ionizer off** **(<600 s)**	**Ionizer on** **(600 - 2800 s)**	**Ionizer off** **(>2800 s)**	

Ventilator tubing *	770	28800	510	26.6°C & 33.4% RH

Ventilator tubing **	510	77200	480	25.1°C & 40.2% RH

Mask*	460	37100	300	22.3°C & 41.6% RH

Mask **	1650	85600	430	23.3°C & 40.2% RH

Nebulizer tubing *	1230	84600	360	23.3°C & 41.7% RH

Nebulizer tubing **	480	52200	340	23.5°C & 41.3% RH

Unometer™ (measuring chamber) *	450	65500	240	23.9°C & 39.8% RH

Unometer™ (measuring chamber) **	520	74300	240	24.4°C & 38.4% RH

Unometer™ (tubing) *	800	58600	360	23.7°C & 42.8% RH

Unometer™ (tubing) **	390	56800	320	24.2°C & 40.3% RH

Disposable apron *	820	54300	670	24.3°C & 37.8% RH

Disposable apron **	640	47800	420	24.3°C & 37.3% RH

* in free space

** in contact with table

**Figure 3:**
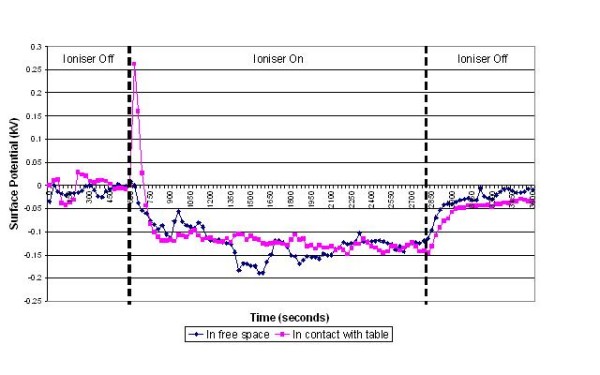
**Change in surface potential over time for ventilator tubing in the presence of negative air ions**.

**Figure 4:**
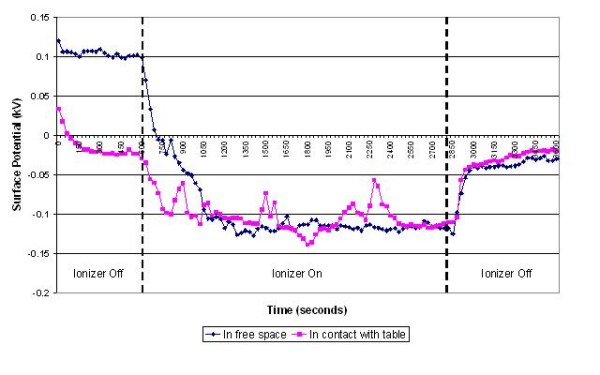
**Change in surface potential over time for the face mask in the presence of negative air ions**.

**Figure 5:**
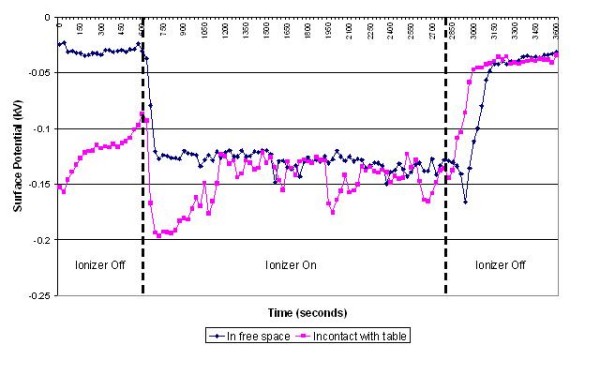
**Change in surface potential over time for nebulizer tubing in the presence of negative air ions**.

**Figure 6:**
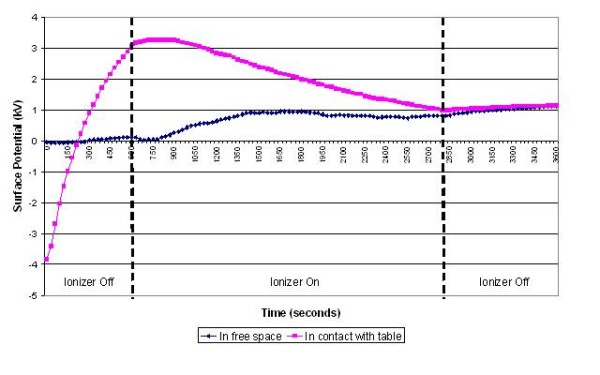
**Change in surface potential over time for the Unometer™ measuring chamber in the presence of negative air ions**.

**Figure 7:**
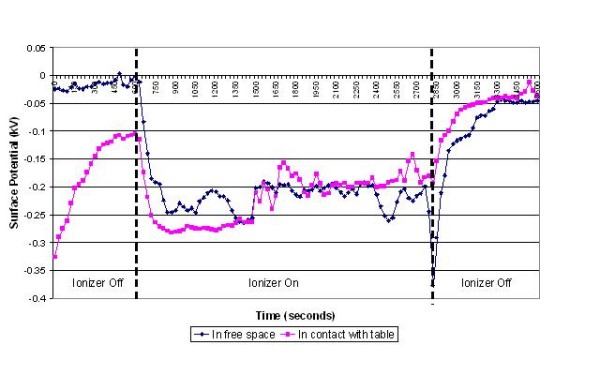
**Change in surface potential over time for the Unometer™ tubing in the presence of negative air ions**.

**Figure 8:**
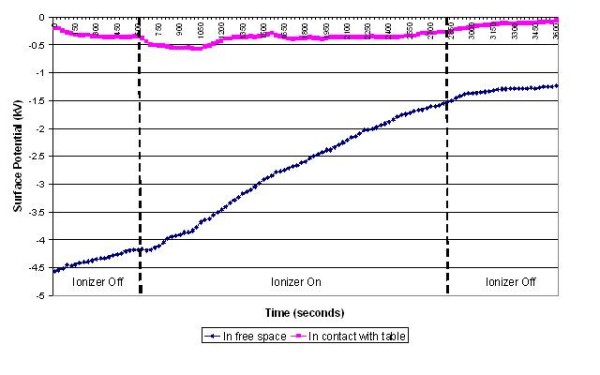
**Change in surface potential over time for the disposable apron in the presence of negative air ions**.

The results of the experiments on the various items of equipment are presented in figures 3, 4, 5, 6, 7 and 8 below. These show sequential plots of the potential recorded on the surface of the various specimen items. From these, it can be seen that, with the exception of the disposable apron (Figure 8) and the Unometer™ plastic measuring box (Figure 6), the other items of medical equipment all behaved in a similar fashion when the ionizer was in operation - they all rapidly developed a large negative charge (i.e. generally >-100V) in the presence of the ionizer. However once the ionizer was switched off, all these specimens quickly lost their large negative charge and returned to a steady slightly negative (<-50V) potential (figures 3, 4, 5 and 7). Whether or not the item of equipment was in contact with the earthed table appeared to matter little - in both cases the negative potential developed during ionization was approximately the same. In the case of the nebulizer and Unometer™ urinary tubing, when these items of equipment were in contact with the table there appears to have been a gradual leaking of charge to earth.

The results obtained for the disposable apron and the Unometer™ measuring chamber were somewhat different from those for the other items of equipment. When suspended in free space the Unometer™ chamber acquired a positive surface potential in the region 700 - 1000 V, which remained after the ionizer was switched off. However when the box was in contact with the table, the action of the ionizer steadily reduced the very high initial positive potential to a level similar to that recorded when the Unometer™ box was in free space. As with the free space experiment, when the ionizer was switched off the surface potential of the box remained steady at approximately +1000 V. These data suggest that the polymer used to construct the box has radically different triboelectric properties to the polymer used in the Unometer™ urinary tubing. By comparison, the results for the polyethylene apron appear less consistent. When in free space, the action of the ionizer on the apron was similar to that on the Unometer™ box - that is, the action of the ionizer caused the surface potential of both items of equipment to become more positive. Whereas when the apron was in contact with the table, the action of the ionizer initially promoted a strong negative surface potential (>-500 V), which then stabilized at approximately -350 V. However, after the ionizer was switched off, the apron rapidly lost this negative potential.

## Discussion and conclusion

Numerous studies have implicated contamination of the clinical environment with outbreaks of *Acinetobacter *associated infection. For example, in a study conducted in an ICU, multiple-antibiotic-resistant *Acinetobacter *spp. isolates were recovered from bed surfaces, surfaces of equipment, floor mops (when dry) and curtains [18]. Pulsed field gel electrophoresis typing revealed the patients' isolates and those from the environment to be indistinguishable. Other studies have implicated the aerial dissemination of *Acinetobacter *spp. in the transmission of infection. Allen and Green [17] were the first to suggest airborne dissemination of *Acinetobacter*-carrying particles. Investigating an outbreak of multiply-antibiotic-resistant *A. anitratus *in an ICU, a medical ward and three neurosurgical wards, they cultured the outbreak strain from 16 of 82 settle plates. Das *et al*. [18] hypothesized that heavily contaminated bed curtains when moved would promote the airborne spread of *Acinetobacter *spp. Weernink *et al*. [19] investigated airborne dispersal of *Acinetobacter *spp. from patient's pillows. Using settle plates they found aerial dissemination from feather pillows, but not from synthetic pillows. Further evidence is provided by Houang *et al*. [21] who placed 70 settle plates in an ICU and 120 (in total) in four surgical wards. Remarkably, 96% of plates in the ICU and 89% in the surgical wards were culture-positive, demonstrating widespread airborne dispersal. Gerner-Smidt [22] recovered an outbreak of strain *A. calcoaceticus *subsp. *anitratus *from the air in an ICU using both settle plates and a slit sampler. Others have also shown *Acinetobacter *spp. to be readily culturable from hospital air [23,24].

In the clinical setting bacteria are readily liberated into the air through activities such as bed making and curtain shaking [15,25]. Furthermore, large numbers of bacteria (i.e. >750 bacteria per minute) may be aerially disseminated from individuals undertaking activities within the clinical environment [26]. The charge carried by these airborne bacteria can be very high indeed, and is generally much greater than that carried by inert particles in the air [27,28]. This suggests that bacteria have inherently charged surfaces. Indeed, studies on waterborne bacteria indicate that they can carry thousands of elementary charge units [29]. If highly charged airborne bacteria pass through an electric field generated by a plastic object, then they are likely to move either towards, or away, from the surface depending on the polarity of the charges involved. Indeed, Allen *et al*. [20] in a study of plastic items of medical equipment, demonstrated that such equipment frequently becomes charged during routine activities (without the presence of ionizers) to such an extent that it attracts airborne bacteria.

Given that *Acinetobacter*-carrying particles are present in the air in many clinical settings, the electrostatic characteristics of the environment are likely to have a profound effect on their deposition. The data in figures 3, 4, 5, 6, 7 and 8 suggest that negative air ionizers, if installed on a ward, are likely to significantly alter the surface potential of many items of plastic equipment, provided there is a sufficient ion generation rate within the ward space [11,30]. The results presented here indicate that the charge depends on the triboelectric properties of the material. However, it is probably that most non-conducting items of plastic equipment, such as ventilator and nebulizer tubes will take on a negative charge, while some other items may become positively charged. The particles in the air will also become predominantly negatively charged through a combination of field and diffusion charging [11], with the result that they will be repelled from negatively charged surfaces and attracted to positively charged or earthed materials.

From the results presented above it can be seen that in the presence of the ionizers most of the items of equipment developed a significant negative charge (i.e. in the region -100 to -200 V). For example, for the 25 mm diameter LDPE ventilator tube, which achieved an average potential of -124 V and a capacitance of 14 pF when in contact with the table, it can be calculated that the charge developed is 1.736 × 10^-9 ^C. Therefore, using equation 1 and the data presented in Table 1, it can be calculated that an electric field of strength 42493 V/m exists around the ventilator tube. If an airborne particle containing a unit charge (i.e. one additional electron) enters the electric field around the ventilator tube it will be repelled by a force of 6.81 × 10^-15 ^N, which equates to a terminal velocity in the region 1.3 to 5.2 mm/s for an 8 μm particle, depending on its density. Given that when negative air ionizers are in operation, the vast majority of airborne particles will gain a negative charge, it is clear that an electrostatic repulsive force of this magnitude would ensure that many small to medium sized aerosol particles (1-8 μm) will be deflected from the surface of the tube, with the result that surface contamination will be minimized. Given that in reality there will be other forces at play due to local air velocity, further studies coupling electrostatic effects with room airflows using simulation techniques are underway [11]. Initial results have shown that the extent to which repulsion or attraction occurs depends on particle size, ion generation rate and the magnitude of charge - thus supporting the findings presented here that suggest sufficient charge can be developed by an ionizer to change the deposition pattern on items of equipment with a relatively small surface area such as ventilator tubes. This may explain why the action of the negative air ionizers in Kerr *et al'*s study [1] was associated with increased deposition of *Acinetobacter*-carrying particles on bed frames and VDU screens. If high charges are accumulated, then it is possible that even relatively substantial particles such as large skin squamae, that would otherwise settle out, could be repelled from sensitive surfaces.

*Acinetobacter *respiratory tract infections have been frequently associated with contamination of ventilators respiratory therapy equipment, including nebulizers. For example, Craven *et al*. [31] found that out of 19 nebulizers tested, 79% were contaminated predominantly with *Acinetobacter*, *Pseudomonas *and *Klebsiella *spp. and that 71% of these generated bacterial aerosols with the resulting droplet nuclei of <3 μm, capable of penetrating the distal airways of the lungs. It was found that the nebulizers had become contaminated by reflux from the patients mixing with condensate in the ventilator circuit. In another ventilator associated outbreak [32] contaminated ventilator tubing and humidifiers were identified as the source of infection. It was found that decontamination of the equipment was not occurring due to the action of a faulty washing machine. Replacing the reusable tubes with disposable tubing ended the outbreak. Dealler [33] reported an unusual outbreak of *A. baumannii *infection in an ICU involving the failure of the bacterial filter separating the patient from the ventilation tubing, with the result that outbreak strain was detected in the air near to the output ducts of the ventilation machines. Twenty-six of these filters were cultured and in 15 cases *Acinetobacter *had colonized the condensate on the patient side of the filter and could also be detected by swabbing on the equipment side, indicating failure of the filters. In addition, the outbreak strain was recovered from various parts of the ICU, including some locations untouched by the staff, suggesting that airborne dissemination of *A. baumannii *was taking place.

In our experiments the ventilator, nebuliser and urinary tubes (figures 3, 5 and 7) all exhibited similar behaviour when the ionizer was switched on. They all rapidly became negatively charged, which is not surprising given that these items of equipment are made from either polyethylene (PE) or polyvinylchloride (PVC), both of which are strongly negative in the triboelectric series [34,35] and therefore likely to gain electrons. The SealFlex™ mask (Figure 4) also behaved in a similar manner, suggesting that its triboelectric properties are similar to those of PE and PVC. Interestingly, all these items of equipment rapidly lost their negative charge once the ionizer was switched off. This phenomenon could have been due to bulk conduction, or alternatively charge loss may have occurred through the recombination of electrons with positive ions in the air [36]. The results in Figure 8 show that the polyethylene disposable apron, when in contact with the earthed table, performed in a similar manner to the ventilator, nebuliser and urinary tubes. However, when suspended in free space its behaviour was completely different, with its surface potential becoming more positive when the ionizer was in operation. The reasons for this are unclear.

From Figure 6 it can be seen that the data obtained for the Unometer™ measuring chamber were very different to those from the other items of equipment. This appears to be because this item was manufactured from styrene acrylonitrile (SAN), which is a much more 'positive' triboelectric material than either PE or PVC. Styrene acrylonitrile, like polystyrene (PS), is a polymer with a high electrical resistivity, which can hold either a positive or negative electrical charge for hours [37]. This probably explains why the Unometer™ box retained a positive charge of approximately 100 V after the ionizer had been switched off.

Although the impact of corona discharges on polymers has been investigated by other researchers [36,38], to our best knowledge this is the first study of its kind to specifically examine the subject in a clinical context. As such, our results provide a plausible explanation for the observations of Kerr *et al*. [1] in their study of *Acinetobacter *infection/colonization on an ICU. Our findings suggest that it is possible that the action of negative air ionizers in this setting changed the electrostatic characteristics of plastic items of equipment within the ICU environment, causing airborne particles to be strongly repelled from some surfaces or attracted to others. This is wholly consistent with the observations of Kerr *et al*., who found a marked increase in environmental isolates of *Acinetobacter *spp. to be associated with the operation of the ionizers. If this hypothesis is indeed the case, then it would suggest that observations of Kerr *et al *in their ICU-based study were related to the electric field created by the ionizers in the ICU and its subsequent effect on plastic devices rather than any direct antibacterial effect on *Acinetobacter *species.

## Competing interests

The authors declare that they have no competing interests.

## Authors' contributions

SJS, CBB, CJN and PAS designed the study. CFS and SJS undertook the experimental work. KGK advised on the clinical aspects of the study. CBB wrote the manuscript with major contributions from other authors. All authors have read and approved the final manuscript.

## Pre-publication history

The pre-publication history for this paper can be accessed here:

http://www.biomedcentral.com/1471-2334/10/92/prepub
